# Molecular map of disulfidptosis-related genes in lung adenocarcinoma: the perspective toward immune microenvironment and prognosis

**DOI:** 10.1186/s13148-024-01632-y

**Published:** 2024-02-11

**Authors:** Fangchao Zhao, Lei Su, Xuefeng Wang, Jiusong Luan, Xin Zhang, Yishuai Li, Shujun Li, Ling Hu

**Affiliations:** 1 Department of Thoracic Surgery, The Second Hospital of Hebei Medical University, Shijiazhuang, 050000 Hebei People’s Republic of China; 2 Department of Radiation Oncology, Affiliated Hospital of Hebei University, Baoding, 071000 Hebei People’s Republic of China; 3 Pulmonary and Critical Care Medicine, Affiliated Hospital of Hebei University, Baoding, 071000 Hebei People’s Republic of China; 4 Department of Thoracic Surgery, Hebei Chest Hospital, Shijiazhuang, 050000 Hebei People’s Republic of China; 5 Department of Medical Oncology, Hebei Key Laboratory of Cancer Radiotherapy and Chemotherapy, Affiliated Hospital of Hebei University, Baoding, 071000 Hebei People’s Republic of China

**Keywords:** Disulfidptosis, Lung adenocarcinoma, Molecular subtypes, Tumor microenvironment, Immune checkpoint inhibitors

## Abstract

**Background:**

Disulfidptosis is a recently discovered form of programmed cell death that could impact cancer development. Nevertheless, the prognostic significance of disulfidptosis-related genes (DRGs) in lung adenocarcinoma (LUAD) requires further clarification.

**Methods:**

This study systematically explores the genetic and transcriptional variability, prognostic relevance, and expression profiles of DRGs. Clusters related to disulfidptosis were identified through consensus clustering. We used single-sample gene set enrichment analysis and ESTIMATE to assess the tumor microenvironment (TME) in different subgroups. We conducted a functional analysis of differentially expressed genes between subgroups, which involved gene ontology, the Kyoto encyclopedia of genes and genomes, and gene set variation analysis, in order to elucidate their functional status. Prognostic risk models were developed using univariate Cox regression and the least absolute shrinkage and selection operator regression. Additionally, single-cell clustering and cell communication analysis were conducted to enhance the understanding of the importance of signature genes. Lastly, qRT-PCR was employed to validate the prognostic model.

**Results:**

Two clearly defined DRG clusters were identified through a consensus-based, unsupervised clustering analysis. Observations were made concerning the correlation between changes in multilayer DRG and various clinical characteristics, prognosis, and the infiltration of TME cells. A well-executed risk assessment model, known as the DRG score, was developed to predict the prognosis of LUAD patients. A high DRG score indicates increased TME cell infiltration, a higher mutation burden, elevated TME scores, and a poorer prognosis. Additionally, the DRG score showed a significant correlation with the tumor mutation burden score and the tumor immune dysfunction and exclusion score. Subsequently, a nomogram was established for facilitating the clinical application of the DRG score, showing good predictive ability and calibration. Additionally, crucial DRGs were further validated by single-cell sequencing data. Finally, crucial DRGs were further validated by qRT-PCR and immunohistochemistry.

**Conclusion:**

Our new DRG signature risk score can predict the immune landscape and prognosis of LUAD. It also serves as a reference for LUAD's immunotherapy and chemotherapy.

**Supplementary Information:**

The online version contains supplementary material available at 10.1186/s13148-024-01632-y.

## Introduction

Lung adenocarcinoma (LUAD), comprising 60% of all lung cancers, is a prevalent subtype of non-small cell lung cancer (NSCLC) and has emerged as a leading global cause of cancer-related deaths [1–3]. Despite being a highly aggressive form of cancer, recent advances in diagnosis and treatment have markedly enhanced patient outcomes [4]. Nonetheless, the overall survival rate for lung adenocarcinoma patients remains low [5]. Lung adenocarcinoma management depends on factors such as disease stage, scope, and the patient's overall health. Common treatment options for this condition include surgery, radiation therapy, chemotherapy, targeted therapy, and immunotherapy [6]. Patients with similar clinicopathological features exhibit significant variations in drug responses, indicating that traditional TNM staging alone is inadequate for predicting patient outcomes accurately [7, 8]. To resolve this problem, we must identify new signature molecules capable of efficiently categorizing LUAD patients into distinct subgroups, increasing their potential for responding to targeted therapeutic interventions.

The pathogenesis and progression of LUAD are closely related to the imbalance of various cell death mechanisms, such as apoptosis, necrosis, autophagy, and ferroptosis [9]. Recently, a new form of cell death has been discovered: disulfidptosis, which operates independently of the existing programmed cell death processes. Disulfidptosis is a type of cell death induced by disulfide stress due to an excessive accumulation of cystine in the cell, resulting in actin collapse [10]. *SLC7A11* is an important protein that mediates cystine transport. When the expression of *SLC7A11* is elevated, a state of glucose deprivation impedes the production of *NADPH* by the *PPP*, an extensive build-up of small molecule disulfides occurs, causing a cascade of redox abnormalities and cellular apoptosis [11]. Researchers have discovered that treatment with glucose transporter inhibitors (GLUT inhibitors) in preclinical models can induce disulfidptosis in tumors with high expression of *SLC7A11*, such as LUAD [11]. This effectively inhibits tumor growth and provides new insights and strategies for cancer treatment. However, as the study of disulfidptosis is still in its early stages, its role in cancer progression and therapy requires further investigation.

We conducted a thorough bioinformatics analysis of disulfidptosis-related genes (DRGs) in LUAD using publicly available datasets. We assessed their expression patterns, tumor microenvironment (TME) infiltration, prognostic significance, and potential molecular mechanisms in LUAD. Our findings offer new insights into comprehending the molecular foundation of disulfidptosis in LUAD and its impact on diagnosis and treatment.

## Materials and methods

### Data acquisition

Gene expression data and relevant clinical information of LUAD samples were obtained from the publicly available Gene Expression Omnibus (GEO) and The Cancer Genome Atlas (TCGA) datasets. After excluding LUAD samples with incomplete survival time information, a total of 397 LUAD samples and 567 samples (including 507 LUAD samples and 60 normal samples) were chosen from the GSE72094 and TCGA-LUAD datasets, respectively.

The "limma" script was used to convert the transcriptome matrix of TCGA-LUAD from fragments per kilobase million (FPKM) to transcripts per million (TPM). The "sva" package was used to correct the batch effect and normalize the transcriptome matrix of LUAD samples from both the GSE72094 and TCGA-LUAD datasets, and the resulting cohort was named "merge-cohort." Three independent datasets, specifically GSE31210, GSE50081, and GSE68465, were acquired from the GEO database for external validation.

### Unsupervised clustering for DRGs

We extracted 14 DRGs from the previous study [10–14] and provided comprehensive gene information in Additional file 2: Table S1. Using the R package "ConsensusClusterPlus," we conducted consensus unsupervised clustering analysis based on DRG expression levels to classify patients into distinct clusters related to disulfidptosis (referred to as DRG clusters). We conducted principal component analysis (PCA) to illustrate the classification effect of the DRG clusters. Subsequently, we compared the overall survival (OS) probability of the DRG clusters using the R packages "survival" and "jskm."

### Correlations of DRG clusters with chemoradiotherapy sensitivity–related genes (CRSGs), immune checkpoint genes (ICGs), and TME

This study analyzed the expression levels of CRSGs and ICGs in distinct DRG clusters (Additional file 2: Tables S2, S3). To do this, we conducted a comprehensive literature search [15–19] and evaluated the obtained genes to determine their differential expression patterns among distinct DRG clusters. The R package "ESTIMATE" can compute TME scores, which include the stromal score, immune score, and estimate score. The assessment of immune cell infiltration in the TME of LUAD was carried out using the single-sample gene set enrichment analysis (ssGSEA) algorithm.

### Gene set variation analysis (GSVA) and gene set enrichment analysis (GSEA)

To explore the difference of the biological function among DRG clusters, we employed the R package "GSVA" to conduct a GSVA using the "c2.cp.kegg.v7.5.symbols" and "c5.go.bp.v7.5.symbols" gene sets. The R package "pheatmap" was utilized to effectively visualize the obtained results. GSEA was performed by R package "clusterProfiler." The cutoff point of significance was |normalized enrichment score (NES)|> 1, *P*-value < 0.05, false discovery rate (FDR) *Q* value < 0.25 for GSEA.

### Identification of differentially expressed genes (DEGs) between DRG clusters and functional annotation

The DEGs among distinct DRG clusters were identified utilizing the R package "limma." To identify significance DEGs, we set the threshold for |log2(FoldChange)| at > 0.5 and the adjusted *P*-value at < 0.05. To investigate the biological functions of DEGs related to DRG clusters, we performed gene ontology (GO) and Kyoto encyclopedia of genes and genomes (KEGG) enrichment analyses using the "clusterProfiler" package.

### Identification of disulfidptosis gene clusters in LUAD

We conducted univariate Cox regression analysis on DEGs associated with DRG clusters to identify DEGs related to OS (OS-related DEGs). Based on the expression levels of OS-related DEGs, we conducted consensus unsupervised clustering analysis using the R package "ConsensusClusterPlus". LUAD patients were stratified into distinct disulfidptosis gene clusters, and OS time was compared using Kaplan–Meier (K-M) analysis.

### Development of prognostic signature and independent prognosis analysis

At the outset, patients in the merged cohort were randomly split into a training cohort and a testing cohort in a 1:1 ratio using the R package "caret." We utilized the least absolute shrinkage and selection operator (LASSO) Cox regression to reduce the dimensionality of the high-dimensional dataset. This was accomplished by using the R package “glmnet” for the analysis of DEGs related to OS. We conducted a multivariate Cox regression analysis to identify candidate genes, and subsequently subjected them to individual gene expression-based GSEA. Afterward, we proceeded to develop a predictive model for disulfidptosis within the training cohort. We calculated the DRG score for each sample using the following formula:

$${\text{DRG}} - {\text{Score = }}\sum\limits_{i = 0}^{n} {{\text{Coefi}} \times {\text{Xi}}}$$

Here, "Coefi" represents the regression coefficient, and "Xi" represents the relative expression level of gene i. Patients in the training, test, and merge sets were separately divided into low-risk and high-risk groups based on their median risk score. The K-M analysis was conducted separately in the three sets to predict the OS of both the high-risk and low-risk groups. Moreover, the model's accuracy was evaluated through various methods, including the receiver operating characteristic curve (ROC), the C-index, nomograms, and calibration curves. These processes were carried out using R packages in R software version 4.2.1, primarily including “survival,” “survminer,” “timeROC,” “rms,” and “regplot.”

### Comprehensive analysis of the DRG score in LUAD

The CIBERSORT algorithm was employed to assess the abundance of 22 immune cell populations across two DRG score groups in the TCGA dataset. Subsequently, Spearman correlation coefficient was used to explore the possible relationship between the level of infiltrating immune cells and DRG score. The study utilized the ESTIMATE algorithm to evaluate the estimated score, stromal score, and immune score for each sample. We used the “maftools” R package to extract mutation annotation format (MAF) data from the TCGA database in order to analyze the mutational profile of LUAD patients across different DRG score cohorts. We assessed every LUAD patient in the entire TCGA cohort to determine their TMB score. We employed the tumor immune dysfunction and exclusion (TIDE) algorithm to estimate the immunotherapeutic response of individuals diagnosed with LUAD. This algorithm has the potential to help healthcare professionals identify patients who are ideal candidates for immunotherapy.

### scRNA-seq data processing

The scRNA-seq dataset GSE146100 was retrieved from the GEO database. The study encompassed measurements from 10,996 patient cells. The R package "Seurat" was used to analyze gene expression data for individual samples. The filtering criteria were as follows: genes detected in fewer than 3 cells were excluded; cells with fewer than 200 detected genes were excluded; cells with over 10% mitochondrial gene expression were excluded. The expression profiles were first normalized with the Log Normalization algorithm and then further normalized using a linear regression model. We selected the top 2000 highly expressed and variable genes for PCA analysis to identify significant and influential dimensions. We applied the UMAP algorithm to reduce the dimensionality and cluster the cells. We used well-known cell markers from the literature to annotate the cell clusters. We analyzed and visualized the communication network between cells using the R package “CellChat.”

### Exploration of the mRNA and protein expression levels of the seven signature genes

We compared the expression levels of signature genes between LUAD tumor tissues and normal tissues using the TCGA and Genotype-Tissue Expression (GTEx) databases. Additionally, we utilized the Human Protein Atlas (HPA) database (https://www.proteinatlas.org/) to investigate protein expression levels.

### Cell culture and qRT-PCR analysis

The LUAD cell lines (A549, H1299, and HCC827) and the human normal bronchial epithelial cell line (BEAS2B) were generously provided by the Cell Repository of the Chinese Academy of Sciences in Shanghai, China. All cell lines were cultured in RPMI-1640 medium with 10% fetal bovine serum (FBS), 100 U/mL of streptomycin, and 100 U/mL of penicillin at 37 °C in a 5% CO_2_ atmosphere.

We used 1 mL of TRIzol® to isolate total RNA from cell lines, and cDNA was synthesized using reverse transcriptase from the avian medulloblastoma virus and random primers following TAKARA's instructions. SYBR Premix Ex Taq II (Takara, Shiga, Japan) was used for qRT-PCR. Data were analyzed using the 2^−ΔΔCT^ method. The primer sequences are provided in Additional file 2: Table S4.

#### Statistical analysis

We performed the statistical analyses using R software (version 4.0.1), as previously described in this study. The significance level was set at *P* < 0.05, indicating statistical significance.

## Results

### Landscape of genetic and transcriptional variations of DRGs

**Fig.1:**
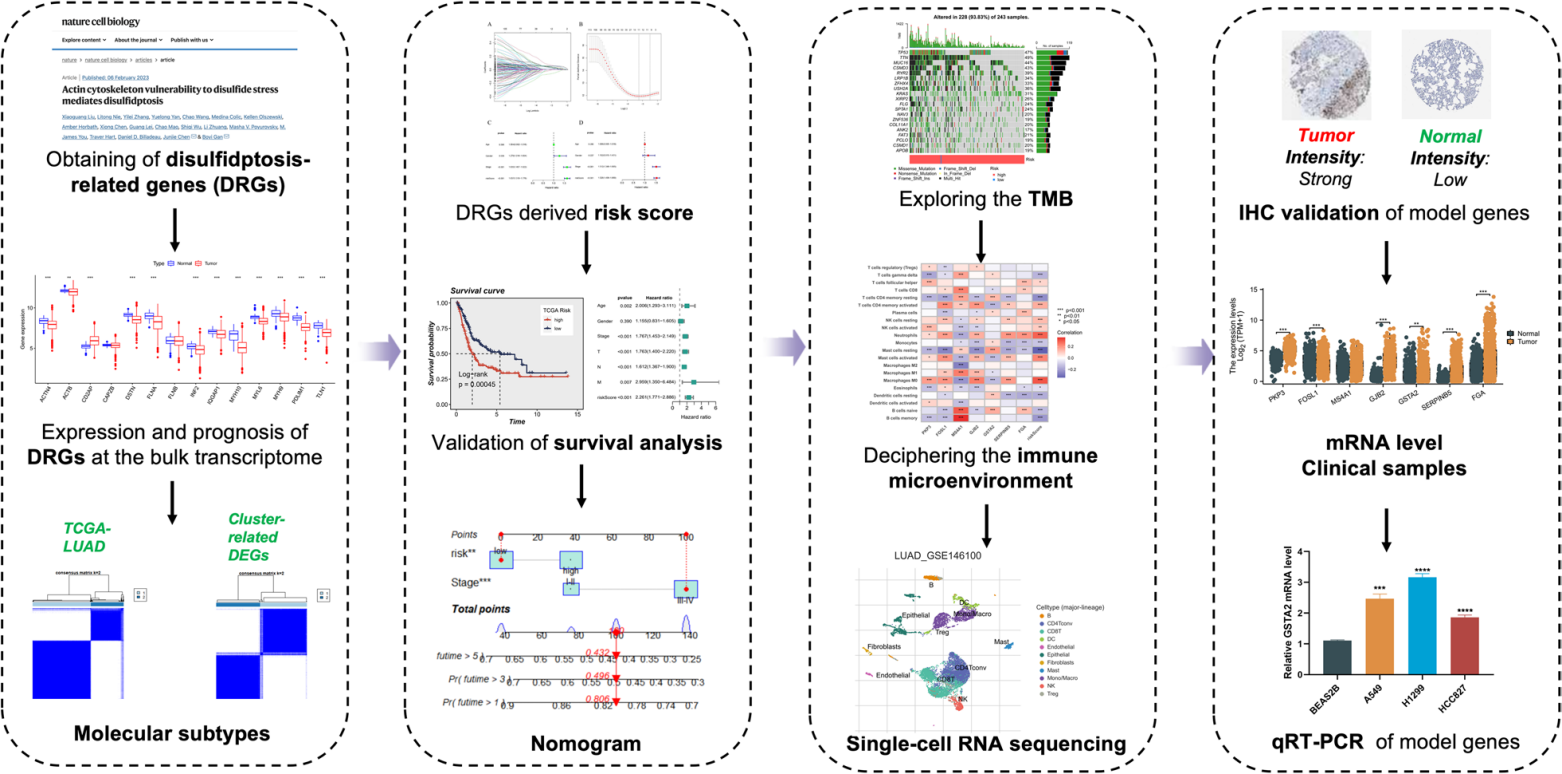
Workflow of this study

**Fig.2:**
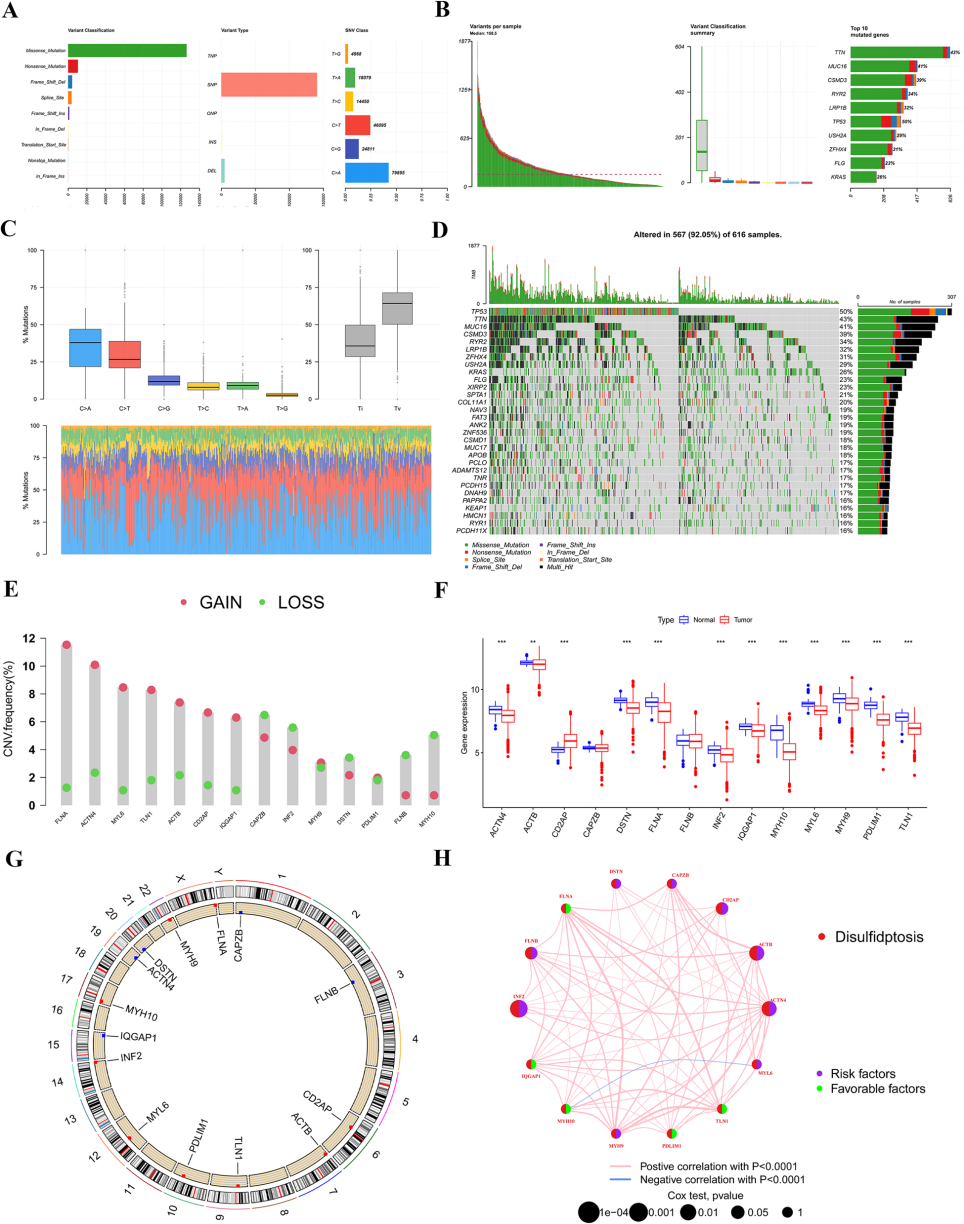
Landscape of genetic and transcriptional variations of DRGs in LUAD. **A, B** Summary of the variation patterns observed in 616 patients with LUAD, including the classification and type of genetic variations, SNV classification, frequency of occurrence of mutations in each sample, and the top 10 most frequently mutated genes. **C, D** Landscape of genetic variations of 616 LUAD patients in TCGA cohort.** E** CNV amplifications and deletions of DRGs in LUAD patients. **F** Variations in the gene expression levels of 14 DRGs in tumor samples compared to their normal counterparts. **G** The circus plot depicted the spatial distribution of CNV in DRGs across 23 chromosomes. **H** The observed network revealed the interconnections between different DRGs in LUAD. In node connections, red indicates positive correlation, while blue signifies negative correlation. The node's size represents the *P*-value of the prognosis, its color indicates the gene's risk—purple for high-risk and green for low-risk. **P < 0.01, ***P < 0.001

Our study's workflow was depicted in Fig. 1. Our study involved a thorough analysis of 14 different DRGs. We visually present the genetic mutation landscape observed in LUAD patients in Figs. 2A–D. Among the 616 patients in the TCGA cohort with LUAD, 567 individuals (92.05%) were discovered to have genetic mutations. Of these mutations, *TP53* had the highest mutation frequency at 50%, followed by *TTN*, *MUC16*, *CSMD3*, and *RYR2*. We examined the copy number variation (CNV) frequencies of 14 DRGs in LUAD. *FLNA* displayed the highest amplification frequency, whereas *CAPZB* and *INF2* exhibited a widespread CNV loss frequency (Fig. 2E). Figure 2G illustrates the locations of CNV alterations in 14 DRGs across 23 chromosomes. Next, we investigated the expression levels, molecular interactions, and prognostic significance of the 14 DRGs. Eleven DRGs, including *ACTN4*, *ACTB*, *DSTN*, *FLNA*, *INF2*, *IQGAP1*, *MYH10*, *MYL6*, *MYH9*, *PDLIM1*, and *TLN1*, were downregulated in tumor samples (*P* < 0.001), while only *CAD2P* was upregulated (*P* < 0.01) (Fig. 2F). The molecular interactions among DRGs are displayed in Fig. 2H.

### Correlations of DRG clusters with clinical features, CRSGs, ICGs, and TME

**Fig.3:**
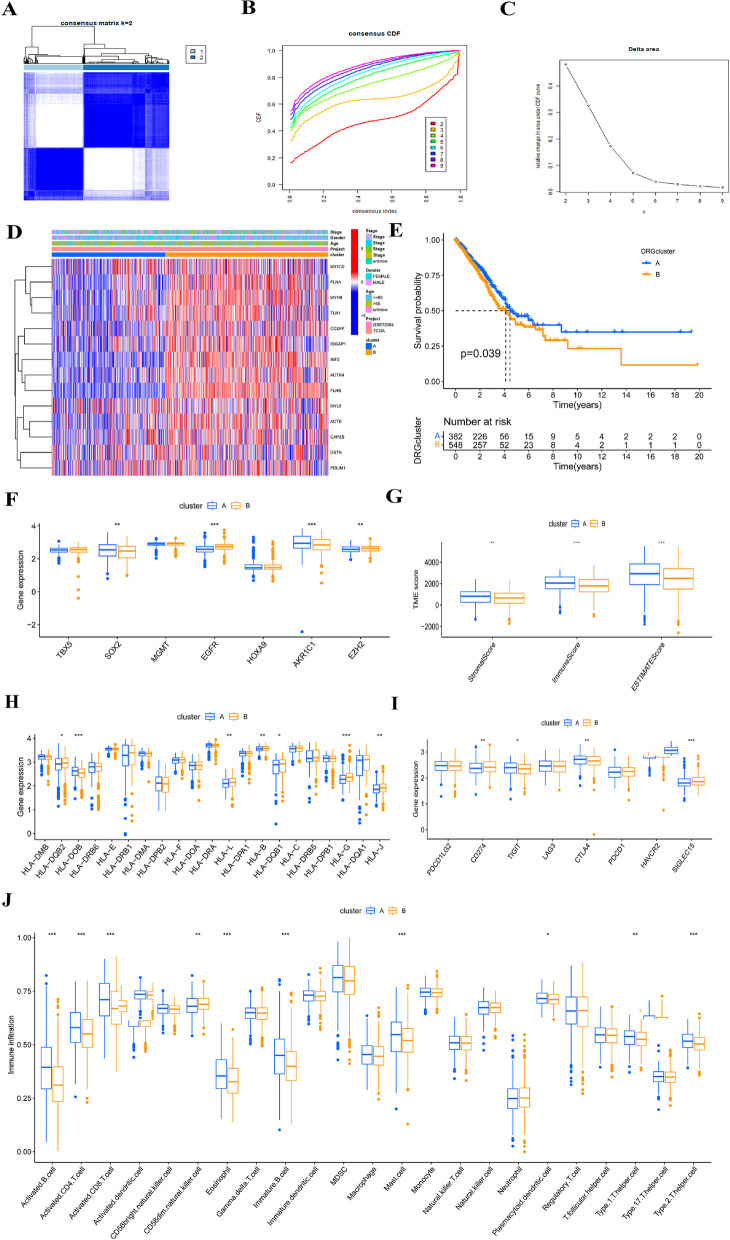
The associations between DRG clusters and clinical features, CRSGs, ICGs, and TME. **A** TCGA-LUAD cohort was grouped into 2 clusters according to the consensus clustering matrix (*k* = 2). **B** Uniform clustering CDF with k from 2 to 9. **C** The change of area under CDF curve with k from 2 to 9. **D** The heatmap demonstrated distinctive expressions of DRGs in relation to clinicopathological characteristics, distinguished DRG cluster A from B. **E** Survival analysis of two DRG clusters using landmark methodology. **F–J** ICGs, immune and stromal scores, MHC molecules expression level, CRSGs, and immune cell infiltration between DRG cluster A and B. *P < 0.05, **P < 0.01, ***P < 0.001

We used a consensus clustering algorithm to classify LUAD patients in the merge-cohort and investigate the expression patterns and underlying biological properties of DRGs. Patients were classified into two distinct DRG clusters, namely DRG cluster A (*n* = 403) and DRG cluster B (*n* = 580), based on the expression of 14 DRGs (Fig**. **3A-C).

Figure 3D displays the distinct expressions of DRGs and clinicopathological characteristics between DRG clusters A and B. We discovered that gene cluster B exhibited a correlation with elevated gene expression levels. Using K-M analysis, we compared clinical outcome differences between DRG clusters (Fig. 3E). The results indicated that patients in DRG cluster B experienced a poorer OS compared to those in DRG cluster A (*P* = 0.039). Additionally, we observed the expression of MHC molecules, ICGs, and CRSGs and identified differential expression among various DRG groups (Figs. 3F, H, I). To explore the potential role of DRGs in immune cell infiltration in LUAD, we conducted an analysis comparing the abundance of immune cells and the TME score between two distinct DRG clusters in the merge-cohort. TME scores were significantly higher in patients categorized in DRG cluster A than in DRG cluster B (Fig. 3G). We also noted significantly higher levels of immune cell infiltration in DRG cluster A compared to DRG cluster B, including activated B cells, activated CD4 + T cells, activated CD8 + T cells, eosinophils, immature B cells, mast cells, and Type 2 T helper cells. Additionally, significantly lower levels of CD56dim natural killer cell infiltration were observed in DRG cluster A compared to DRG cluster B (Fig. 3J).

### Identification of DEGs between DRG clusters and functional annotation

**Fig.4:**
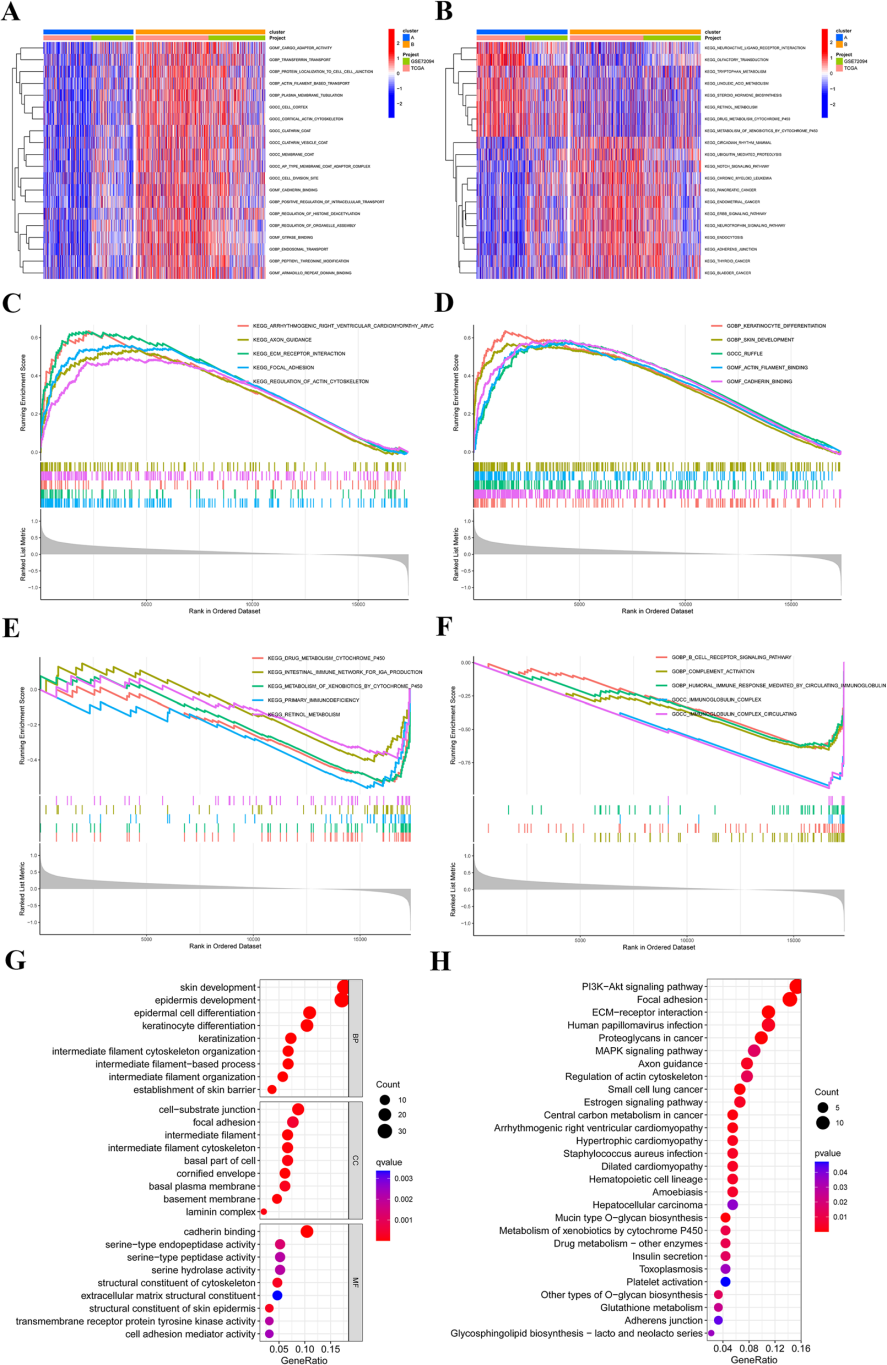
Functional enrichment analysis and discerning DEGs between distinct clusters of DRG. **A** GSVA of KEGG terms between DRG cluster A and B. **B** The GSVA was conducted to assess the differences in GOBP terms between DRG cluster A and B. The color red was assigned to indicate activation, while blue was assigned to indicate inhibition. **C–F** GSEA analysis between DRG cluster A and B. **G, H** GO, and KEGG enrichment analyses of DEGs between two DRG clusters

To delve deeper into the functional annotation between DRG clusters A and B, we performed GSVA and GSEA on the merge-cohort. The GSVA results revealed a significant enrichment of cancer-related pathways, such as pancreatic cancer, endometrial cancer, thyroid cancer, and bladder cancer, in DRG cluster B (Fig. 4A). Moreover, DRG cluster B showed a significant enrichment in processes related to actin transport, including actin filament-based transport, cortical actin cytoskeleton, and the positive regulation of intracellular transport (Fig. 4B). The results from GSEA show that DRG cluster B has a significant association with actin, especially in terms of regulating the actin cytoskeleton and binding to actin filaments (Figs. 4C-F). The "limma" package was used to identify DEGs related to DRG clusters. A total of 198 DEGs were identified, comprising 30 down-regulated genes and 168 up-regulated genes (Additional file 2: Table S5). In line with GSVA and GSEA results, the GO and KEGG analyses indicated associations between these DEGs and actin and cancer (Figs. 4G-H). This supports prior scholarly reports indicating that disulfidptosis is a form of cell death resulting from the disintegration of the actin filament network and is closely linked to tumors [10, 11].

### Identification of disulfidptosis gene clusters

**Fig.5:**
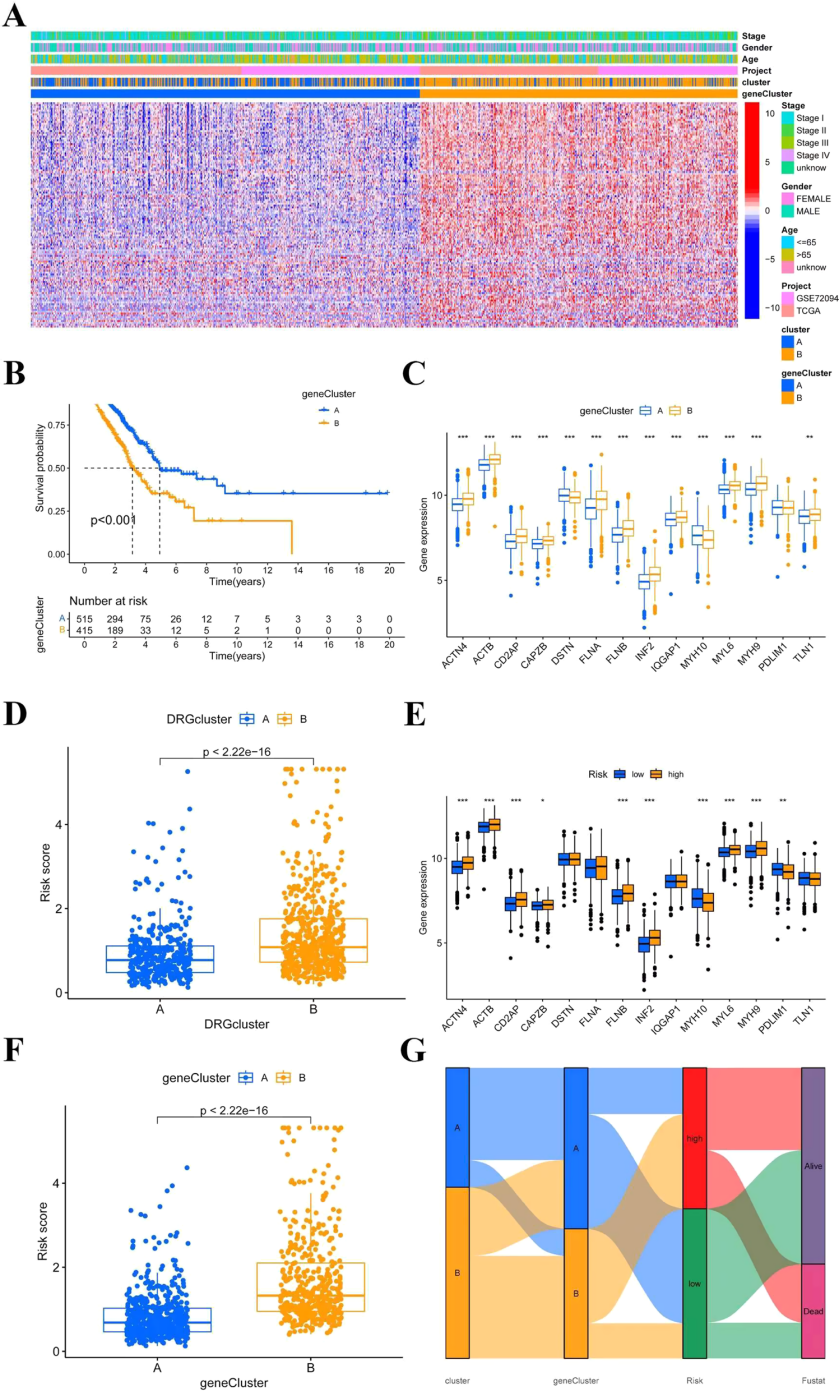
Detection of gene clusters and establishment of the prognostic model associated with disulfidptosis in LUAD. **A** The heatmap displayed distinctive patterns of expression for OS-related DEGs across various gene clusters and clinicopathological characteristics. **B **K-M OS curves for patients in the two gene clusters (log-rank test). **C** Variations in the expression levels of 14 DRGs within distinct gene clusters. **D** The DRG score significant dissimilarities between DRG clusters A and B. **E** Variations in the expression levels of 14 DRGs between groups classified as high risk and low risk. **F **Differences in DRG scores among gene cluster A to B. **G** The Sankey diagram depicts the distribution of subtypes in various cohorts classified by their DRG score and survival rates. *P < 0.05, **P < 0.01, ***P < 0.001

Next, we used univariate Cox regression analysis to assess the prognostic significance of the 198 DEGs related to DRG clusters in the merge-cohort. One hundred and twenty-one genes showed a statistically significant association with OS (*P* < 0.05) and were labeled as OS-related DEGs. Utilizing the expression levels of OS-related DEGs, we applied a consensus clustering algorithm to classify LUAD patients into two gene clusters, referred to as gene cluster A (*n* = 541) and gene cluster B (*n* = 442) (Additional file 1: Figs. S1A-H). PCA analysis showed that gene clusters could be clearly identified (Additional file 1: Fig.S1I). Expression profiles and clinical information of OS-DEGs in different gene clusters are displayed in Fig. 5A. K-M analysis demonstrated a significant association between gene clusters A and B and patient prognosis, with those in cluster B exhibiting a poorer outcome compared to those in cluster A (*P* < 0.001) (Fig. 5B). Additionally, the expression levels of DRGs varied between two distinct gene clusters, with gene cluster B exhibiting higher expression levels than gene cluster A (Fig. 5C).

### Construction and evaluation of the disulfidptosis-related prognostic model

A prognostic model for disulfidptosis was developed through LASSO and multivariate Cox regression analyses on 121 DEGs associated with OS. The merge-cohort was divided into two distinct groups, specifically the training cohort and the test cohort, in a 1:1 ratio. In the training cohort, LASSO and multivariate Cox regression analyses were conducted, leading to the identification of 7 key genes, which include *PKP3*, *FOSL1*, *MS4A1*, *GJB2*, *GSTA2*, *SERPINB5*, and *FGA* (Additional file 1: Figs. S2A-B). We calculated the DRG score based on the coefficients and expression of seven key genes involved in the DRG_S_ (Additional file 1: Fig. S2C). DRG score = 0.202615 × *PKP3* + 0.090719 × *FOSL1* + (-0.10456) × *MS4A1* + 0.09655 × *GJB2* + (-0.0954) × *GSTA2* + 0.083903 × *SERPINB5* + 0.095806 × *FGA*. Univariate and multivariate Cox regression analyses showed that the DRG score can serve as an prognostic factor independent of clinical characteristics (Additional file 1: Figs. S2D-E).

We investigated the relationship among DRG clusters, gene clusters, and DRG score in the merge-cohort. We discovered that DRG score in DRG cluster B was significantly higher than those in DRG cluster A. Additionally, the expression of DRGs was upregulated in the high-risk group, suggesting an association between high DRG score and increased DRG expression with tumorigenesis and actin (Figs. 5D, E). Meanwhile, the expression level of DRG score in gene clusters showed that B was greater than A (Fig. 5F). The Sankey diagram showed subgroup distributions in groups with different DRG score and survival outcomes (Fig**. **5G).

**Fig.6:**
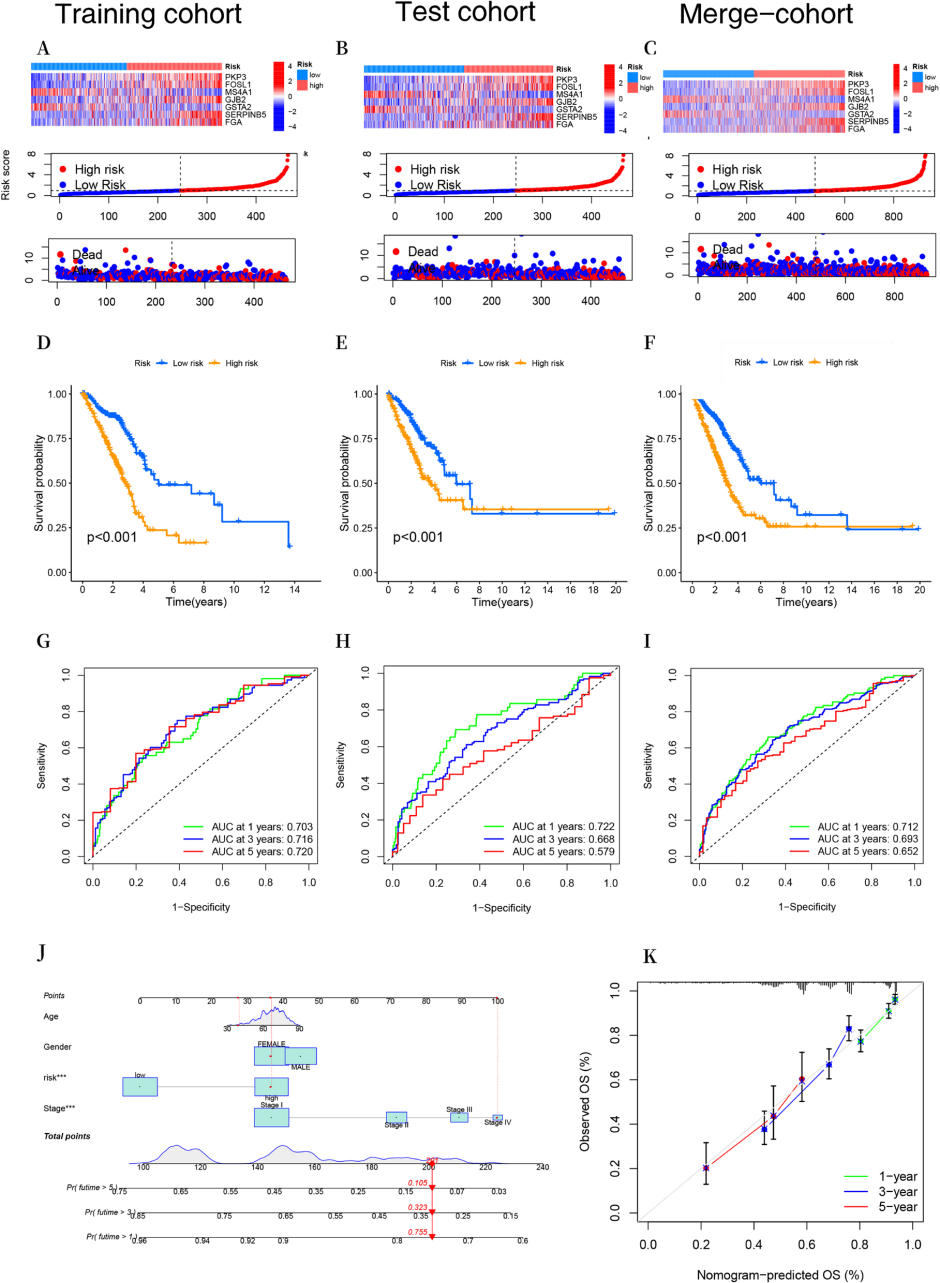
Assessment of the prognostic model associated with disulfidptosis. **A** The heatmap exhibited distinct gene expression patterns in the prognostic model for both high- and low-risk groups across the training, test, and merge-cohorts. **B** The distribution of the DRG score across the training, test, and merge-cohorts. **C** The risk point plot effectively portrayed the survival time and survival status patterns observed within the high-risk and low-risk groups across the training, test, and merge datasets. **D–F** The log-rank test was utilized to analyze the K–M OS curves of patients categorized into high- and low-risk groups across the training, test, and merge-cohorts. **G–I** The prognostic capacity of the prognostic model was evaluated in the training, test, and merge-cohorts using ROC curves. **J** A nomogram was developed to estimate the likelihood of overall survival at 1, 3, and 5 years for patients with LUAD in the merge-cohort. **K** The calibration curves for the nomogram. ***P < 0.001

The thermal map displayed gene expression levels for seven genes in both the high-risk and low-risk groups within the training, test, and merge-cohorts (Fig. 6A). Additionally, an evaluation of the risk plot of the DRG score revealed a significant link between higher DRG score and an elevated mortality rate and shortened survival duration in patients (Figs. 6B, C). The K-M analysis showed a significantly poorer OS for patients in the high-risk group compared to those in the low-risk group across the training, test, and merge-cohorts (*P* < 0.001) (Figs. 6D-F). The AUC values for predicting 1-year, 3-year, and 5-year OS in the training cohort were 0.703, 0.716, and 0.720, respectively (Fig. 6G). The AUC values for predicting 1-year, 3-year, and 5-year OS in the test cohort were 0.722, 0.668, and 0.579, respectively (Fig. 6H). The AUC values for predicting 1-year, 3-year, and 5-year OS in the merge-cohort were 0.712, 0.693, and 0.652, respectively **(**Fig. 6I). A novel nomogram was subsequently developed by combining the DRG score with clinical characteristics in the merge-cohort. This nomogram represents a quantitative method for generating personalized prognostic predictions for LUAD patients (Fig. 6J). Figure 6K shows the calibration curves for 1, 3, and 5-year periods.

To further validate the model's prognostic performance, we utilized three external validation cohorts (GSE31210, GSE50081, and GSE68465). K-M analysis revealed a significantly improved prognosis in the low-risk group compared to the high-risk group (Additional file 1: Figs. S3A, C, E). Additionally, the model exhibited a high AUC value in the external validation cohorts (Additional file 1: Figs. 3B, D, F).

### Correlations of DRG score with TMB and TME

**Fig.7:**
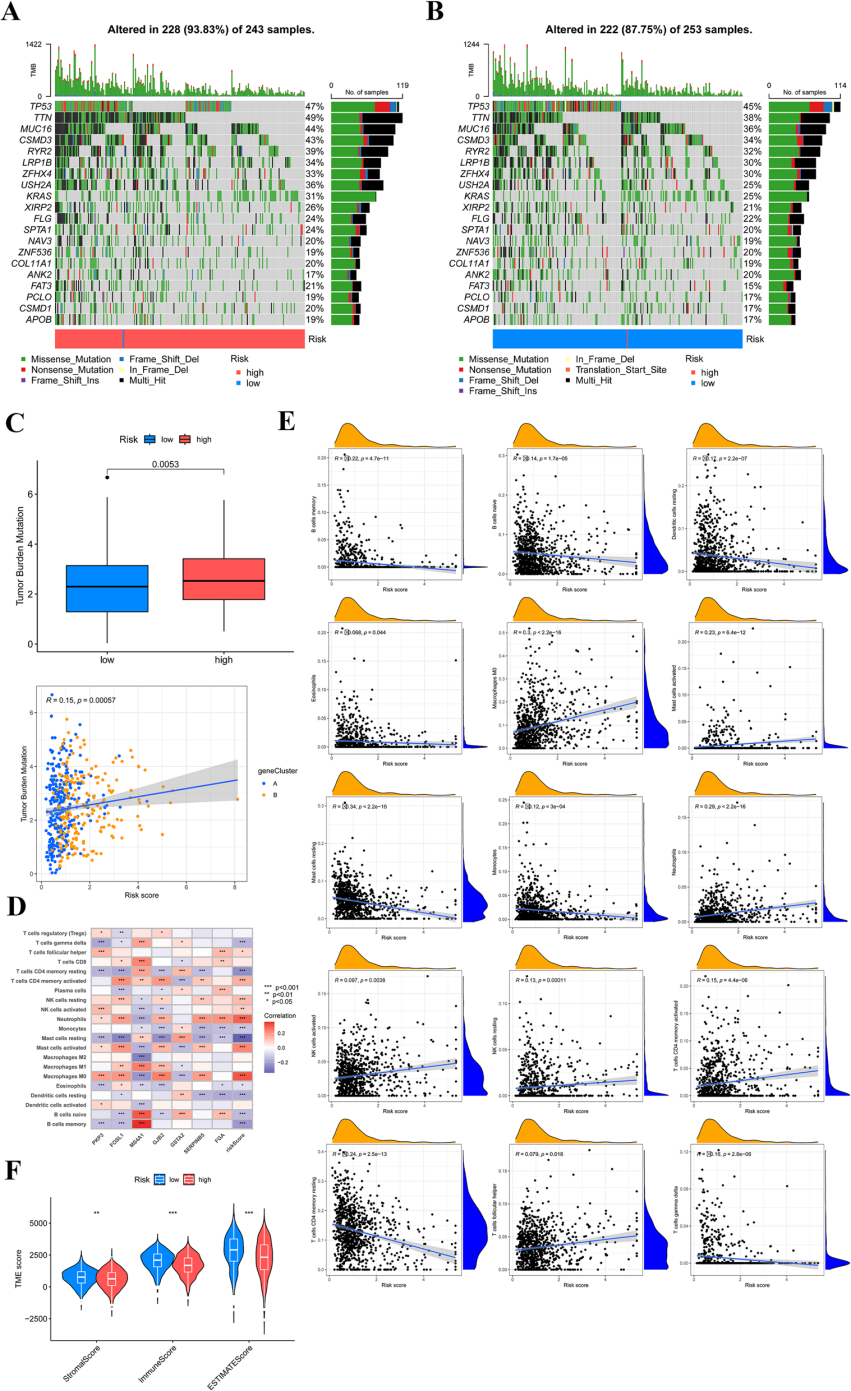
Correlations of the DRG score with TMB and TME. **A, B** The mutational profile of low- and high-risk groups in LUAD patients. **C** The potential correlations that may exist between the TMB and the DRG score across various gene clusters and the discrepancies in TMB score between high- and low-risk groups.** D** The associations between the abundance of immune cells and seven genes in the prognostic model.** E** The association between the prevalence of immune cells and the DRG score.** F** Correlations between DRG score and TME scores. *P < 0.05, **P < 0.01, ***P < 0.001

Previous research has shown a clear link between a higher TMB score and increased response to immunotherapy. The mutational status of different DRG groups was depicted through a waterfall plot in the TCGA dataset. The results showed a notably higher mutation rate in patients from the high-risk group (93.83%) compared to those in the low-risk group (87.75%) (Figs. 7A, B). The group at a higher risk demonstrated a significantly increased TMB score. Additionally, a positive correlation existed between the TMB score and the DRG score (Fig**. **7C). The main goal of this study was to investigate the correlation between immune infiltration and the DRG score, along with the expression levels of seven genes related to the proposed model. Our study's results have revealed a significant connection between the expression of seven genes and most immune cell types (Fig. 7D). Macrophage M0, activated mast cells, activated NK cells, activated T cells follicular helper, activated T cells CD4 memory, and neutrophils exhibited a positive correlation with the DRG score, whereas naive B cells, monocytes, memory B cells, resting mast cells, resting dendritic cells, and resting T cells CD4 memory showed a negative correlation with the DRG score (Fig. 7E). The low-risk group exhibited significantly increased TME scores, comprising the stromal score, immune score, and ESTIMATE score (Fig. 7F).

### Estimation of disulfidptosis-related prognostic model in immunotherapy response

**Fig.8:**
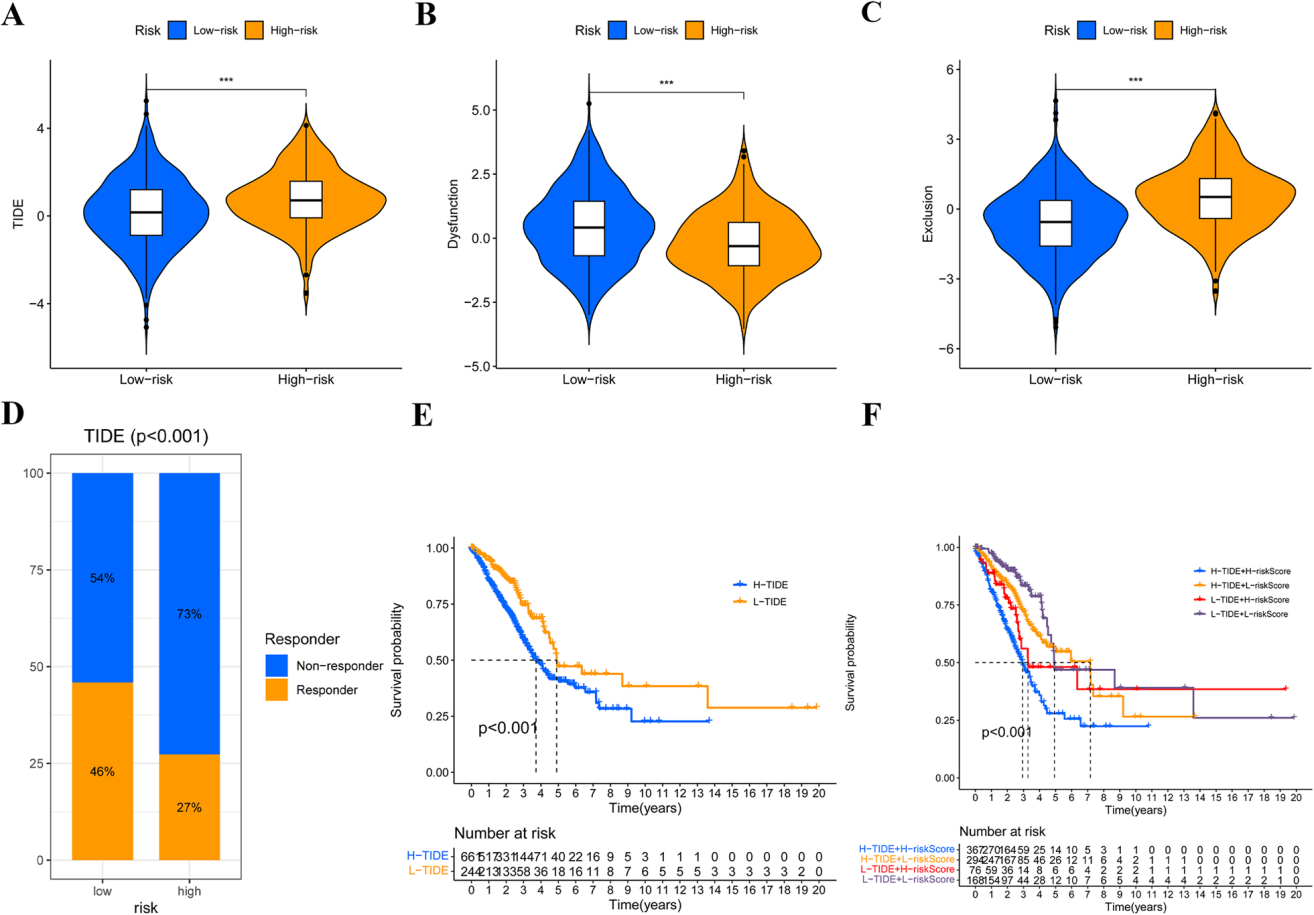
Estimation of the DRG prognostic model in immunotherapy response. **A** A disparity in the TIDE score when compared groups classified as high-risk versus those categorized as low risk. **B** The dissimilarity in exclusion scores observed between groups classified as high risk and low risk. **C** Comparison of dysfunction scores between high- and low-risk groups.** D** The immunotherapeutic response distribution within identified groups has been stratified using the DRG scores derived from the TIDE algorithm. **E** The K-M OS curves were generated to compare two groups categorized based on the TIDE score. **F** The K-M curves were examined to assess the OS of four discrete groups categorized by their DRG and TIDE scores. ***P < 0.001

The TIDE algorithm was employed to assess the response to immunotherapy in LUAD patients, utilizing transcriptomic data from the merge-cohort. The results revealed a significant disparity in TIDE score between high-risk and low-risk groups, with the latter demonstrating a more favorable response to immunotherapy (Fig. 8A). The low-risk group displayed a reduced exclusion score and an elevated dysfunction score (Figs. 8B, C). Furthermore, the TIDE algorithm was utilized to stratify patients into two distinct groups: responders and non-responders. Our research has revealed a significant correlation between a lower DRG score and immunotherapy responders (Fig. 8D). Individuals with elevated TIDE score exhibited poorer prognoses compared to those with lower TIDE score (Fig. 8E). Patients with both high DRG and TIDE score was significantly associated with the worst prognosis (Fig. 8F).

### Tumor microenvironment characterized by single cell sequencing

**Fig.9:**
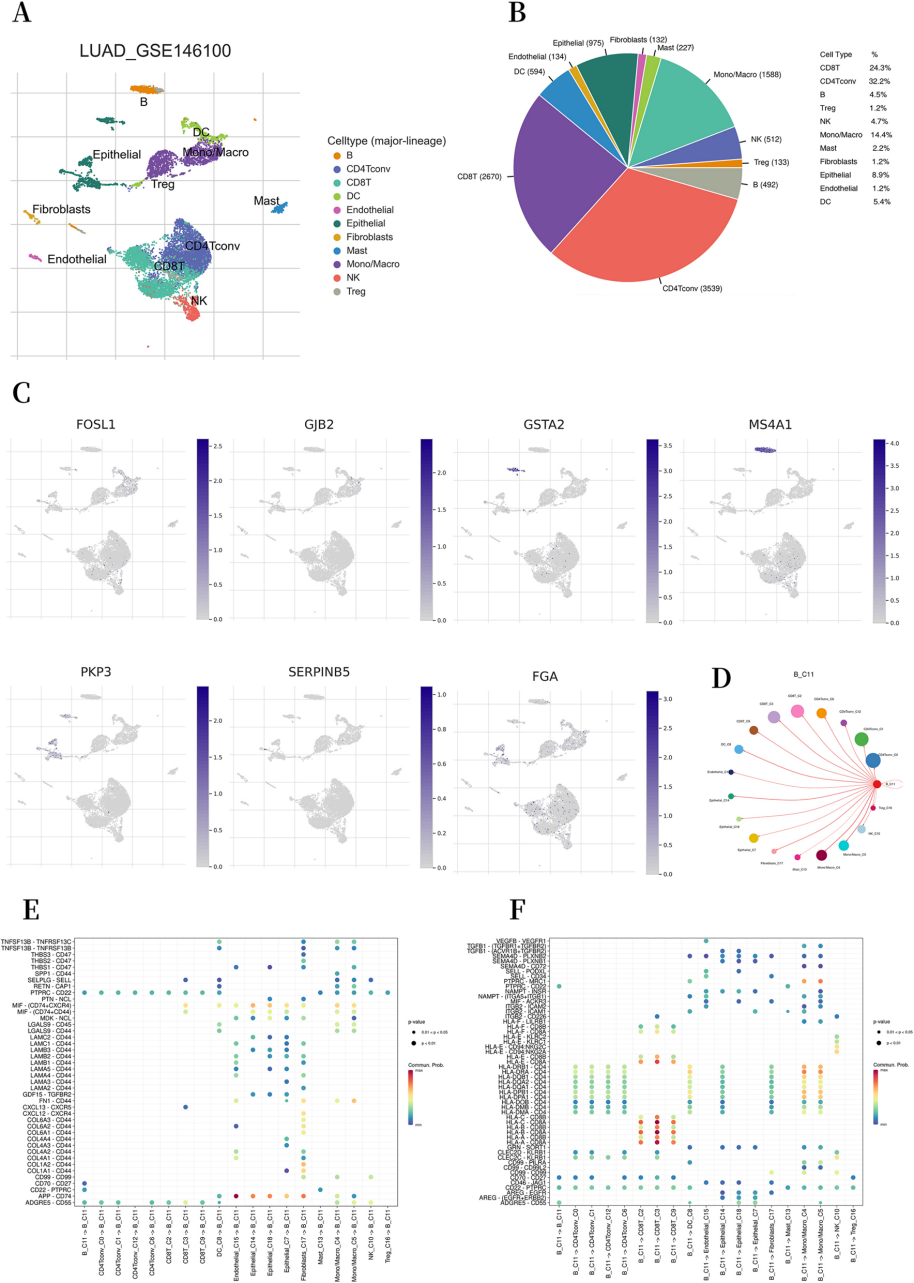
The distribution of the DRG score in tumor microenvironment. **A, B** Eleven cell types from 10,996 cells. **C** Single-cell sequencing analysis has been utilized to investigate the cellular localization of seven modeling genes. **D** Communication between B cells and other cells. **E** Receptor ligand pairs for interactions between B cells and other cell types. **F** Receptor-ligand pairs for interactions between B cells and other cell types. The relative significance of the *P*-value was represented by the size of the circles, with larger circles indicating smaller *P*-value. Additionally, the color of the circles depicted the probability of interactions, with shades of red indicating a higher likelihood of interactions

We used the UMAP algorithm to divide 10,996 cells that had passed quality control measures into eleven cell clusters. Each cluster was labeled based on the expression of specific cell lineage marker genes. Most of the annotated cell clusters were immune cells, including B cells, CD4 + T cells, CD8 + T cells, DC cells, regulatory T cells (Treg), NK cells, and Mono/Macro cells (Figs. 9A, B). To study the expression of model genes across different cell types, we performed an analysis of single-cell sequencing data. The results are shown in Fig. 9C.

*FOSL1* and *GJB2* were mainly expressed in Mono/Macro cells, whereas *GSTA2*, *PKP3*, and *SERPINB5* showed high expression in Epithelial cells. Additionally, FGA was mainly expressed in Epithelial cells, CD8 + T cells, and CD4 + T cells. Interestingly, *MS4A1* was highly expressed in B cells and served as a marker gene for B cells [20]. We conducted a cell communication analysis to investigate how B cells communicate with other cells. The results showed a stronger interaction between B cells and T cells (Fig. 9D). We then conducted a detailed examination of the ligand-receptor pairs involving B cells and other cell types engaged in mutual interactions, using bubble plots. Communication between B cells and other cellular components may be facilitated by receptor-ligand pairs (Figs. 9E, F).

### Validation of the expression and alteration of the seven genes in LUAD tissues

**Fig.10:**
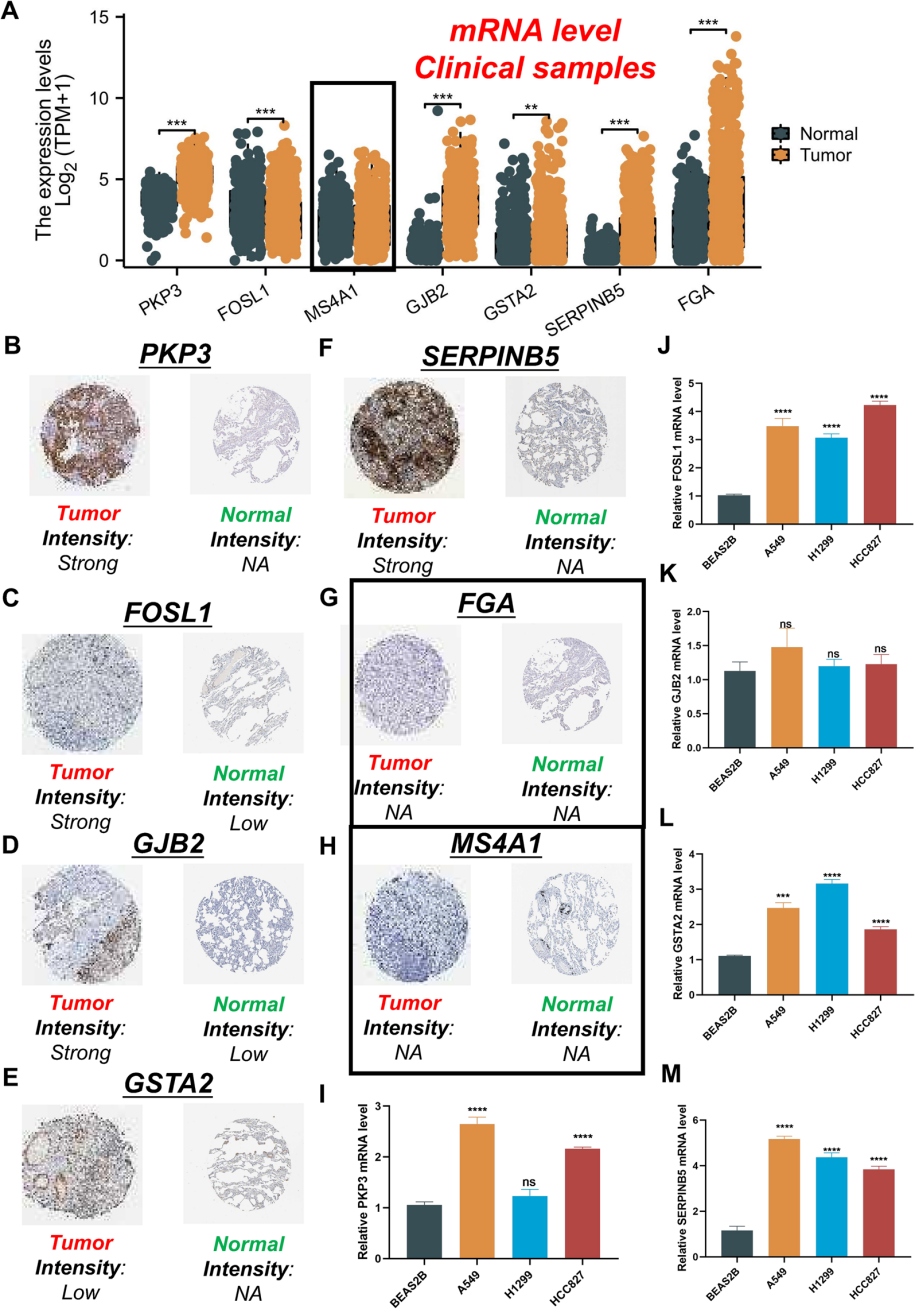
Validation of the expression of the seven signature genes in LUAD. **A **The mRNA expression profile of the seven genes in tumor tissues from the TCGA database and normal lung tissues from the TCGA and GTEx databases. **B-H** The protein expression of the seven genes in LUAD tumor tissues and normal tissues. The data were obtained from the HPA database. **I–M **Further verification of the mRNA expression levels of five signature genes in human LUAD cancer cell lines and human normal lung epithelial cell line by qRT-PCR analysis. ns, not significant, *P < 0.05, **P < 0.01, ***P < 0.001

To investigate the clinical significance of the seven genes in the model, we validated their mRNA expression levels using the TCGA and GTEx databases. As depicted in Fig. 10A, the mRNA expression levels of *PKP3*, *FOSL1*, *GJB2*, *GSTA2*, *SERPINB5*, and *FGA* were significantly elevated in LUAD tissues. *MS4A1* expression showed no difference between the LUAD and normal samples. HPA analysis showed that the protein levels of PKP3, FOSL1, GJB2, and SERPINB5 were significantly upregulated in LUAD tissues compared to normal lung tissue, while the levels of GSTA2 were significantly decreased in LUAD tissues (Figs. 10B-F). MS4A1 and FGA did not exhibit significant staining in both tumor and normal samples (Figs. 10G, H). Moreover, the qRT-PCR results clearly showed that the mRNA expression levels of *PKP3*, *FOSL1*, *GSTA2*, and *SERPINB5* were significantly higher in human LUAD cells compared to normal human lung epithelial cells, while *GJB2* showed no statistically significant difference (Figs. 10I-M). In conclusion, these results provide additional confirmation of the stability and reliability of the risk signature.

## Discussion

Recently, a novel cell death model called disulfidptosis has become a subject of study. This distinctive cell death mechanism is triggered by disulfide stress and represents a unique entity among the extensively studied forms of regulated cell death [10, 21]. In this model of cell death, the excessive accumulation of intracellular disulfide molecules leads to disulfide stress, which, in turn, binds to actin cytoskeleton proteins, ultimately causing the collapse of the actin network and, consequently, cell death [11]. Embracing this concept would undoubtedly improve our understanding of cell death mechanisms and provide a viable therapeutic approach for cancer treatment by specifically mitigating disulfidptosis. Furthermore, Ni and Qi have already outlined the prognostic characteristics of disulfidptosis in LUAD in their studies [22, 23], but it is imperative to enhance their predictive efficacy. Furthermore, there is a significant deficiency in the field of molecular subtypes based on DRGs.

In this study, we comprehensively and systematically examined 14 DRGs at both the genetic and transcriptional levels in the context of LUAD. Using the differential expression levels of 14 DRGs in the TCGA-LUAD dataset, we successfully defined two distinct and robust molecular subtypes closely linked to disulfidptosis, referred to as DRG cluster A and B. In DRG cluster B, there was a significant decrease in overall prognosis, along with a noticeable reduction in the expression levels of ICGs, CRSGs, and TME scores when compared to DRG cluster A. Furthermore, DRG cluster B displayed a marked enrichment in metabolic pathways and processes associated with the cell cycle. Additionally, this specific cluster showed a significant association with both cancer and the actin cytoskeleton. These findings clearly establish that distinguishing DRG-based clusters provides an innovative approach to classify LUAD. Subsequently, 198 DEGs were identified within the two DRG clusters. A comprehensive enrichment analysis of these DEGs revealed their significant connections to both processes related to cancer and the control of actin dynamics. This supports Liu et al.'s findings, suggesting that disulfidptosis is a type of cell death caused by the disruption of the actin filament network, showing a strong connection to oncogenic processes [10, 11].

Multivariate Cox analyses of DEGs between DRG clusters revealed 121 OS-associated DEGs. Similar to the clustering of DRG phenotypes, two gene subtypes were identified based on these DEGs. These subtypes demonstrated a significant relationship with patient prognosis, indicating their predictive potential for LUAD. To more accurately assess the disulfidptosis patterns in individual LUAD patients, we developed a predictive prognostic model related to disulfidptosis (DRG score system), which consisted of *PKP3*, *FOSL1*, *MS4A1*, *GJB2*, *GSTA2*, *SERPINB5*, and *FGA*. *PKP3* is a widely expressed member of the PKP family that is present in both monolayer and stratified epithelial tissues that contain desmosomes [24]. *PKP3* plays a crucial role in the development and progression of cancer by promoting malignant biological activity and is considered an essential biomarker for early cancer diagnosis and prognosis evaluation [25]. The *FOSL1* protein is commonly regarded as an integral subunit of the *AP1* transcriptional complex. Its function is critical in various cellular processes, including cell differentiation, response to environmental stresses, and tumorigenesis [26]. The gene *MS4A1* encodes CD20, a crucial marker found on the surface of B cells. CD20 plays a significant role in B cell receptor signaling and its interaction with the immune microenvironment. Moreover, *MS4A1* has been shown to have a correlation with the lipid metabolism and immune microenvironment status of individuals with cancer, which suggests its potential as an independent prognostic indicator [27]. Located on chromosome 13q12.11 and comprising three exons, *GJB2* (also referred to as connexin 26) is regarded as an oncogene. Numerous types of cancer have been linked with *GJB2*, which has been shown to promote tumor growth, EMT, and lymph node metastasis [28]. *SERPINB5*, also referred to as Maspin, belongs to the serine protease inhibitor superfamily as a non-inhibitory member. Historically, it has been recognized as a tumor suppressor with the ability to impede cancer cell migration and invasion, as well as to induce apoptosis primarily in cancer models [29]. The insufficiency of *FGA* has been found to have a significant impact on the speed of tumor progression and metastasis in patients with lung cancer and it has been observed to encourage tumor growth and metastasis via the integrin-AKT signaling pathway [30].

Accumulating evidence suggests potential roles for signature genes in LUAD. In our validation experiments, despite heterogeneity in protein-level expressions, almost all genes were significantly validated at the mRNA level. Meanwhile, patients with gene cluster B showed higher DRG score and the worst outcomes, and a higher DRG score is typically associated with increased DRG expression levels in LUAD tissues. Further analysis of the correlation among the DRG cluster, gene cluster, DRG score, and survival status highlighted the robust and stable prognostic-predictive ability of our scoring system. Distribution plots and a K-M plot confirmed that as the DRG score increased in the TCGA training and test cohorts, survival times decreased. This predictive capability was further substantiated by three independent validation cohorts from GEO. Additionally, patients with low- and high-risk groups exhibited significant variations in their responses to radiotherapy and chemotherapy. Additionally, our nomogram demonstrated superior clinical benefits in predicting the prognosis of LUAD patients compared to individual independent prognostic factors. In conclusion, we have successfully demonstrated the independent and predictive role of the DRG score in LUAD.

Recent studies have shown a strong correlation between disulfidptosis and immune infiltration, where high disulfidptosis subtypes demonstrate higher immune scores [10]. Our findings are consistent with these results, as the application of the CIBERSORT algorithm indicated that individuals classified as high-risk showed a tendency toward enhanced anti-tumor immune responses. During our investigation, we observed increased expression levels of crucial anti-tumor immune cell populations, such as NK cells, CD4 + T lymphocytes, and macrophages, in the high-risk group. Our current study has limitations in thoroughly investigating the role of infiltrated immune cells in LUAD. However, there is a research gap in the field regarding the understanding of the involvement of infiltrated immune cells in LUAD. Further research is needed to elucidate the interactions between immune cell types, their abundance, function, and their correlation with tumor development and prognosis in LUAD. Such investigations would enhance our understanding of the immune microenvironment in LUAD and provide a foundation for the development of novel immunotherapeutic strategies. In our current study, we have made preliminary explorations in this area, but further research is warranted. The TIDE analysis results revealed that individuals categorized as low-risk patients exhibited a reduced likelihood of immune evasion. This observation suggests that individuals in this patient cohort may potentially gain greater therapeutic benefits from immunotherapy, while also potentially reducing resistance to ICI.

However, despite the promising results, there are several issues that require attention. Firstly, it is important to note that the DRG risk signature was developed retrospectively using publicly available databases, potentially introducing inherent selection bias. To determine the generalizability and robustness of our results, it is crucial to conduct extensive prospective and multicenter clinical investigations. Additionally, considering the limited availability of therapy-related information, such as surgical interventions, targeted therapies, and immunotherapies, for the majority of patients in public databases, it was not feasible to standardize treatments and assess the predictive efficacy and accuracy of the DRG signature specifically for patients who underwent surgery, received targeted therapy, or underwent immunotherapy. Consequently, this limitation may introduce prognostic biases into the predictions. As a result, their inclusion in future studies is necessary. Lastly, it is still unknown whether these key genes are associated with glucose deprivation and, consequently, serve as an indicator of disulfidptosis. Further comprehensive studies are necessary to investigate the intricate association between this novel form of cell death and tumors.

## Conclusion

In summary, our study offers a comprehensive analysis of DRG expression profiles in LUAD and introduces a novel risk model for assessing therapy response and patient prognosis. The results of this study hold significant clinical significance and indicate that disulfidptosis could potentially serve as a therapeutic target for individuals diagnosed with LUAD.

### Supplementary Information

**Additional file 1. Figure S1: **Unsupervised clustering for OS-related DEGs.** Figure S2: **Construction and verification of prognosis DRG score.** Figure S3: **External validation of the DRG prognostic model in GEO cohort.

**Additional file 2. Table S1: **The list of 14 disulfidptosis-related genes.** Table S2: **The list of 7 immune checkpoint genes.** Table S3: **The list of 8 chemotherapy sensitivity-related genes.** Table S4: **Primer sequences for mRNAs.** Table S5: **The list of 198 DEGs.

## Data Availability

The original contributions presented in the study are included in the article/supplementary material. Further inquiries can be directed to the corresponding authors.

## Abbreviations

LUAD: Lung adenocarcinoma
DRGs: Disulfidptosis-related genes
TME: Tumor microenvironment
GEO: Gene expression omnibus
TCGA: The cancer genome atlas
PCA: Principal component analysis
ICGs: Immune checkpoint genes
CRSGs: Chemoradiotherapy sensitivity-related genes
GSVA: Gene set variation analysis
GSEA: Gene set enrichment analysis
DEGs: Differentially expressed genes
K-M: Kaplan–Meier
OS: Overall survival
LASSO: Least absolute shrinkage and selection operator
ROC: Receiver operating characteristic
TIDE: Tumor immune dysfunction and exclusion
GO: Gene ontology
KEGG: Kyoto encyclopedia of genes and genomes
AUC: Area under the curve
GTEx: Genotype-tissue expression
HPA: Human protein atlas
IHC: Immunohistochemistry
qRT-PCR: Quantitative real-time polymerase chain reaction
