# Supplementary material for: Molecular map of disulfidptosis-related genes in lung adenocarcinoma: the perspective toward immune microenvironment and prognosis
Source: Clin Epigenetics. 2024 Feb 11;16:26. doi: 10.1186/s13148-024-01632-y (PMC10860275; doi:10.1186/s13148-024-01632-y)
Supplement: Supplementary file 1 — Additional file 1. Figure S1: Unsupervised clustering for OS-related DEGs. Figure S2: Construction and verification of prognosis DRG score. Figure S3: External validation of the DRG prognostic model in GEO cohort. [file 13148_2024_1632_MOESM1_ESM.docx]

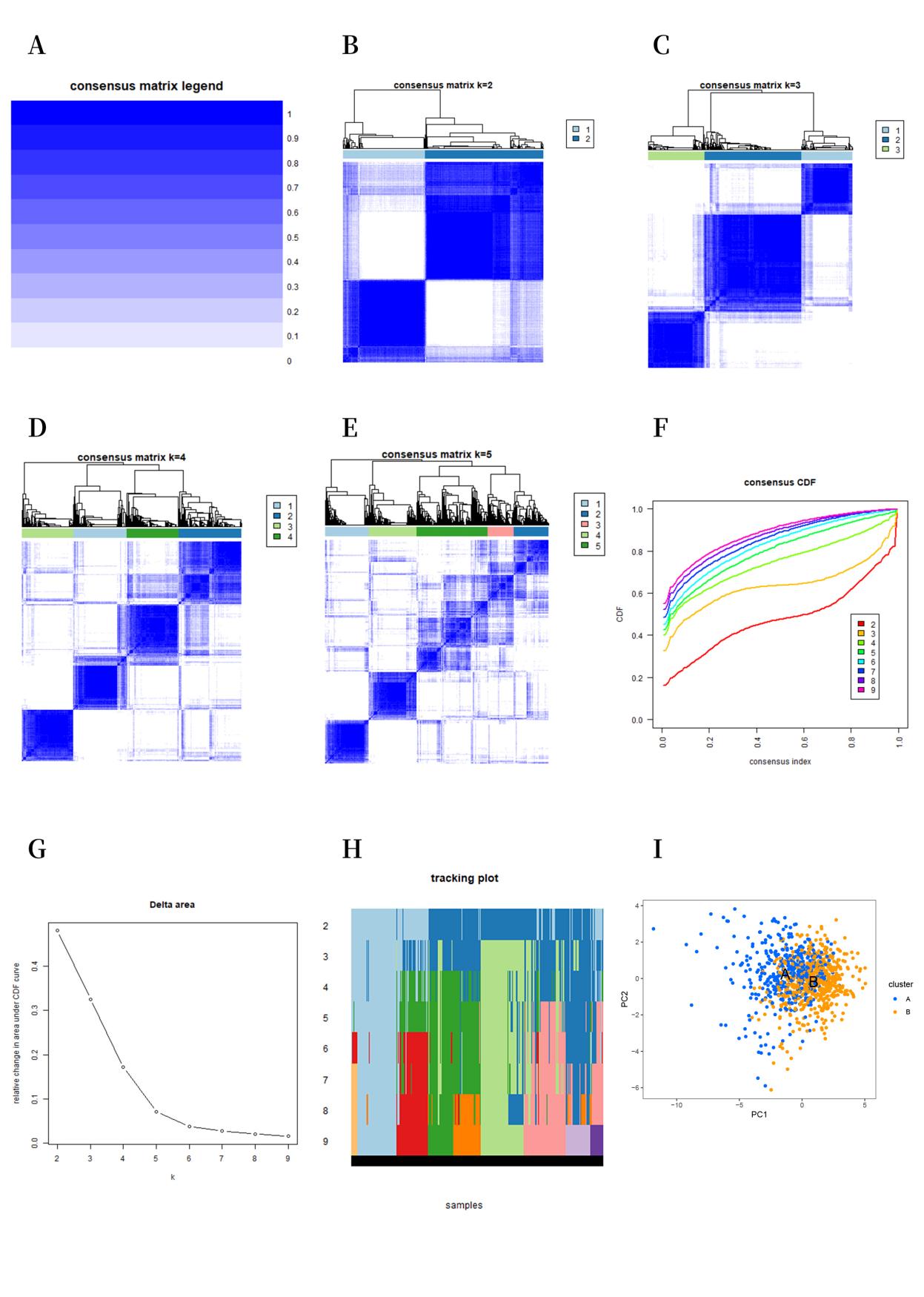


**Supplementary Figure 1. Unsupervised clustering for OS-related DEGs. (A-E)** Merge cohort was grouped into 2 clusters according to the consensus clustering matrix (k = 2). **(F)** Uniform clustering cumulative distribution function (CDF) with k from 2 to 9. **(G)** The change of area under CDF curve with k from 2 to 9. **(H)** The tracking plot showed the relationship between samples and clusters. **(I)** The PCA plot showed the distribution among 2 gene clusters.


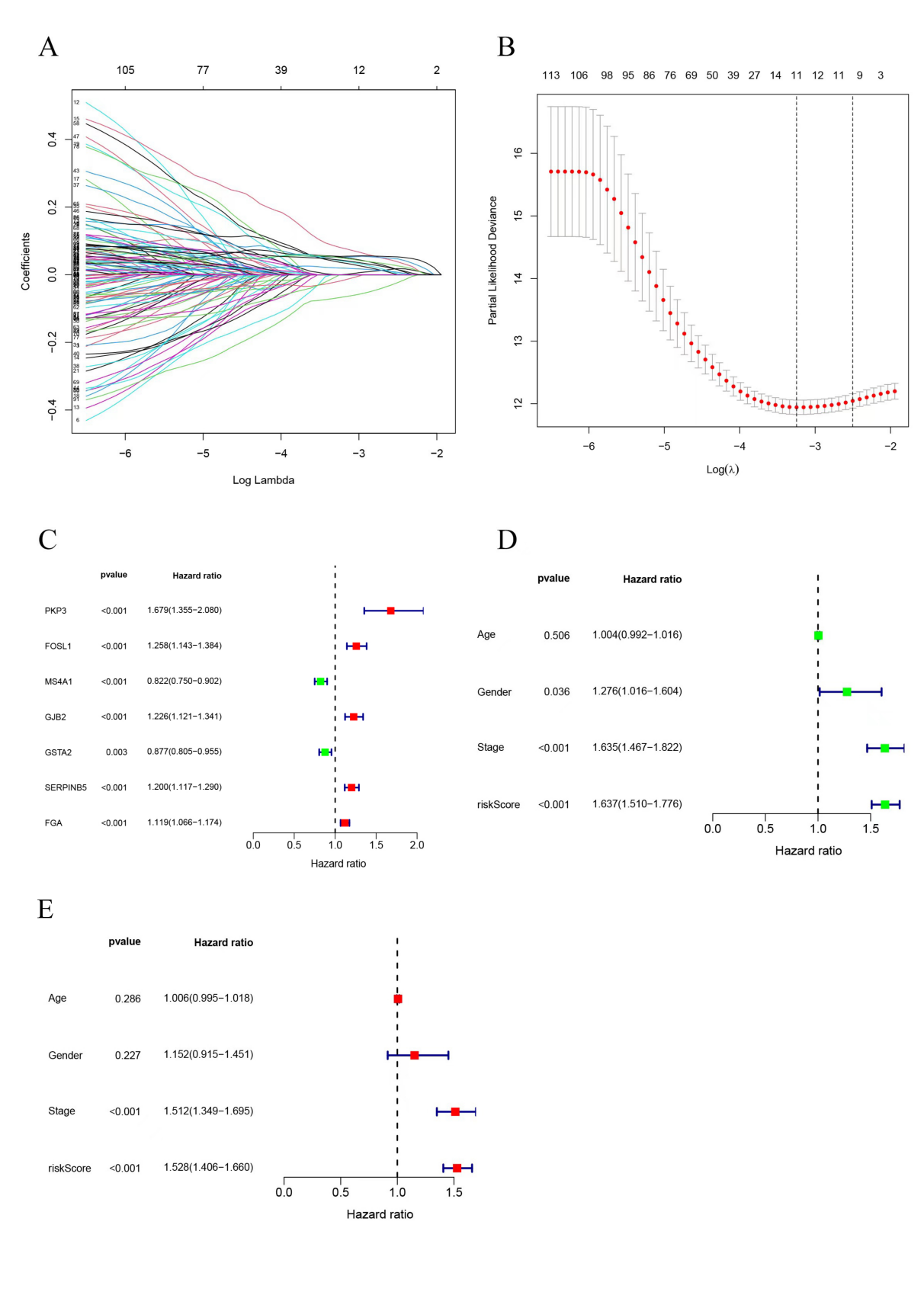


**Supplementary Figure 2. Construction and verification of prognosis DRG score. (A, B)** LASSO coefficient profiles of OS-related DEGs and cross-validation for tuning the parameter selection in the LASSO regression. **(C)** Multivariate Cox regression analysis of DRGs was shown by forest plot. **(D, E)** Univariate and multivariate Cox regression analyses of DRG score and clinical characteristics in merge-cohort.


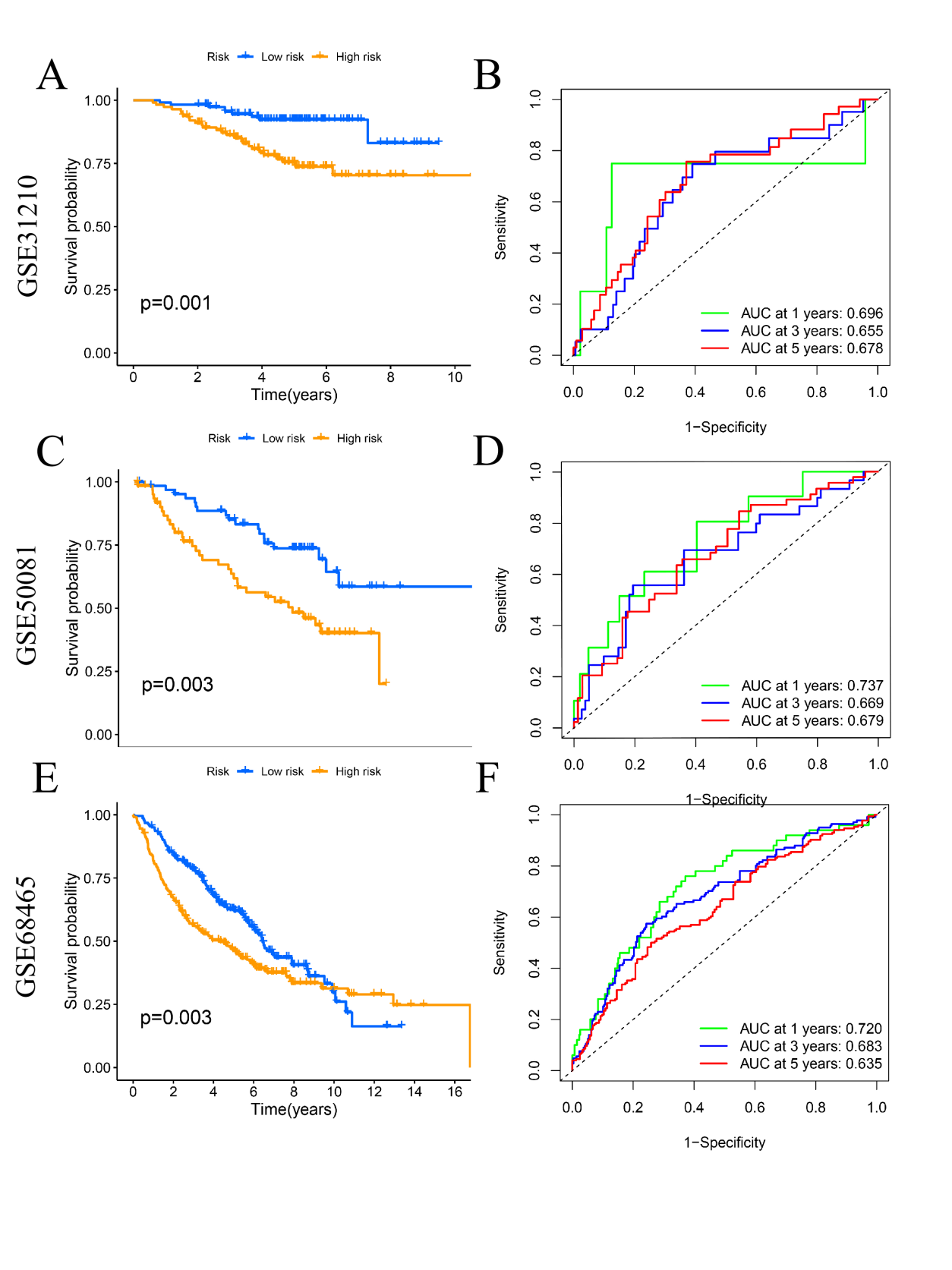


**Supplementary Figure 3**: **External validation of the DRG prognostic model in GEO cohort.**

The K-M OS curves for patients in the high- and low-risk groups in the GSE31210 **(A)**, GSE50081 **(C)**, and GSE68465 cohorts **(E)**. ROC curves showed the prognostic performance of the DRG prognostic model in the GSE31210 **(B)**, GSE50081 **(D)**, and GSE68465 cohorts **(F).**
